# Molecular markers of resistance to sulphadoxine-pyrimethamine during intermittent preventive treatment of pregnant women in Benin

**DOI:** 10.1186/1475-2875-10-196

**Published:** 2011-07-19

**Authors:** Gwladys Bertin, Valérie Briand, Diana Bonaventure, Ambre Carrieu, Achille Massougbodji, Michel Cot, Philippe Deloron

**Affiliations:** 1 Institut de Recherche pour le Développement (IRD), Mère et enfant face aux infections tropicales (UMR216), Faculté de Pharmacie, 4 avenue de l'Observatoire, 75270 Paris Cedex 06, France; 2 Université Paris Descartes, Paris, France; 3 Laboratoire de parasitologie, Faculté des Sciences de la Santé (FSS), Cotonou, Bénin; 4 Centre d'Étude et de Recherche sur le Paludisme Associé à la Grossesse et l'Enfance (CERPAGE), Cotonou, Bénin

## Abstract

**Background:**

The prevention of malaria faces with the repeated emergence of *Plasmodium falciparum *resistance to drugs, often involving point mutations of the target gene. In the pregnant woman, currently the WHO recommendation is the administration of an intermittent preventive treatment (IPTp) with sulphadoxine-pyrimethamine. Sulphadoxine-pyrimethamine (SP) resistance has increased for several years in Africa, stressing the need for alternative molecules. In this context, the first randomized clinical trial comparing the efficacy of SP and mefloquine for IPTp has been conducted recently in Benin. Using samples from this trial, the current study evaluated and quantified the prevalence of mutations on the *pfdhfr *and *pfdhps *genes as well as the copy number of the *pfmdr1 *gene in parasites from *P. falciparum*-infected pregnant women before first and second IPTp administration, and at delivery.

**Methods:**

PCR-restriction fragment length polymorphism of polymorphic codons of the *pfdhfr *gene (51, 59, 108, and 164) was performed. The identification of mutations in three codons of the *pfdhps *gene (436, 437 and 540) was achieved by PCR and sequencing. Copy number quantification for *pfmdr1 *gene was performed using real-time PCR.

**Results:**

Results show a high prevalence rate of mutant parasites in women taking IPTp with sulphadoxine-pyrimethamine or mefloquine. The prevalence of triple and quadruple mutants was high before first drug regimen administration (79/93, 85%), and remained similar until delivery. Infection with mutant parasites was not correlated with low birth weight nor placental infection. In all samples, the copy number of *pfmdr1 *gene was equal to one.

**Conclusions:**

The clinical trial comparing SP and mefloquine efficacy during IPTp showed SP remained efficacious in preventing low birth weight. The present study shows a high prevalence of triple and quadruple mutations implicated in SP resistance. Although the *pfdhfr*/*pfdhps *triple and quadruple mutations were frequent, there was no evidence of correlation between these genotypes and the lack of efficacy of SP in the context of IPTp. Nevertheless, it is now obvious that SP will soon be compromised in whole Africa. Molecular markers have been recommended to monitor SP efficacy for IPTp, but given the current prevalence of mutant parasites their usefulness is questionable.

## Background

*Plasmodium falciparum *infection during pregnancy is responsible for placental infection, and constitutes a substantial risk for the mother, her foetus, and the neonate. It is a major cause of anaemia and maternal death, and one of the main causes of low birth weight (LBW). The WHO recommends protecting the women during pregnancy using intermittent preventive treatment (IPTp) with one curative regimen of sulphadoxine-pyrimethamine (SP) (1,500 mg sulphadoxine and 75 mg pyrimethamine), at least twice during pregnancy, once during the 2^nd ^trimester, and then at least one month apart. IPTp with SP has proven efficacious in reducing the burden of pregnancy-associated malaria (PAM), and is currently part of the national malaria prevention programme in most African countries.

A recent study has compared three studies performed in Benin, showing that SP-IPTp is effective both in reducing the LBW rate, as compared to chloroquine prophylaxis (10% with SP-IPTp given as national policy and 8.7% in a controlled IPTp trial ([1], vs. 15.7% with chloroquine [2]), and the malaria placental infection prevalence rate (11.2% vs. 2.9% and 16.7%, respectively), with a good overall compliance of the national IPTp [3]. However, resistance to SP is developing increasingly in Africa. Several molecular epidemiology studies showed that resistance to pyrimethamine is associated with the acquisition of mutations in the gene coding for dihydrofolate reductase (*pfdhfr*) (S108N, N51I, and C59R, and I164L) [4,5]. The S108N mutant exhibits a low level of resistance, the N51I/S108N or the C59R/S108N double mutants intermediate levels of resistance, and the N51I/C59R/S108N triple mutant has a higher level of resistance to pyrimethamine. Similarly, resistance to sulphadoxine is due to three mutations in the gene encoding dihydropteroate synthase (*pfdhps*) (S436F, A437G, K540E) [6,7]. Each successive mutation causes a greater decrease in sensitivity respectively to pyrimethamine and sulphadoxine. It has been showed that the increased prevalence of mutations on the *pfdhfr *and *pfdhps *gene is linked to intensive use of SP. Indeed, a study showed that the frequency of the triple *pfdhfr *(N51I, C59R, S108N) and double *pfdhps *(A437G, K540E) mutants increased by 37% - 63% and 200% -300% respectively when SP was used as the first line treatment of malaria attacks [8]. Another study showed that the frequency of *pfdhfr *mutations increased, especially after the change in treatment policy [9].

In many countries, SP now demonstrates inadequate therapeutic efficacy in children under 5 years old, and is no longer the drug of choice for treatment. However, data collected in young children cannot be extrapolated to pregnant women, in whom SP IPTp seems to still retain its efficacy [10,11]. Nevertheless, the reducing activity of SP requires further investigations, especially in the context of IPTp. To date, mefloquine (MQ) is one of the most attractive alternatives to SP for IPTp. A randomized trial comparing the efficacy of SP and MQ for IPTp has recently been completed in Benin [11], showing that SP IPTp kept efficacy in a context of growing resistance to SP [12]. Indeed, SP and MQ were found to be equivalent and highly efficacious in the prevention of LBW.

This study was extended with the aim to search for mutations of the genes associated to SP resistance and to evaluate the copy number of *pfmdr1 *gene during pregnancy and at delivery in Beninese women under IPTp. This exploratory investigation looked at the impact of the point mutations on the outcome of pregnancy.

## Methods

### Study site and subjects

A randomized clinical trial comparing the efficacy of SP and MQ for IPTp was conducted from July 2005 to April 2008 in the rural city of Ouidah, Benin. A total 1,601 women were randomized to receive MQ (n = 802) or SP (n = 799). All enrolled women were followed from their first antenatal care visit at the 2^nd ^trimester of pregnancy until delivery. The treatment consisted of two curative doses of SP (1,500 mg of sulphadoxine and 75 mg of pyrimethamine per dose) or MQ (15 mg/kg per dose). Drugs were administered under observation. At the time of IPTp administration (first and second doses given during the 2^nd ^and 3^rd ^trimesters of pregnancy), and at delivery, blood was collected for thick smear confection and filter paper blotting. Details of this clinical trial have been previously reported [11].

All filter papers corresponding to positive blood smears were analysed for *pfdhfr *and *pfdhps *mutations and *pfmdr1*copy number. At delivery, samples from all women presenting or having presented with at least one positive smear during the follow-up were analysed, even if concomitant thick blood smear was negative.

### DNA extraction and genotyping of point mutations

Blood collected on filter paper spots was dried and conserved at room temperature until DNA extraction using "Qiagen Kit" [13]. Genotyping for mutations in codons 51, 59, 108, and 164 of the *pfdhfr *gene was achieved by a technique of DNA amplification by PCR of 40 cycles with 4 μl DNA extract [14,15]. The amplicon obtained was 718 bp and was submitted to a second PCR of 25 cycles with 1 μl of the first PCR product to frame areas of interest. The fragment size obtained varied between 189 and 412 bp. The PCR products were digested by restriction enzymes that varied with the studied codon (Tsp509I, XMN I, Alu I, and Dra I, respectively). For codon 51, the presence of two DNA bands of 148 bp and 64 bp demonstrates a wild genotype, and contrasts to a single band of 212 bp observed with a mutant genotype. Similarly, for codons 108 and 164, a wild genotype is characterized by two or three bands of 322 to 54 bp and 171, 135, 106 bp, respectively. The mutant genotype exhibits a single band of 376 pb for codon 108, and four bands of 143, 135, 106 and 28 pb for codon 164. Finally at codon 59, a mutant genotype is characterized by two bands of 162 and 27 pb, and a wild genotype by a single band of 189 pb. For each analysis, control strains were used, consisting in both the 3D7 strain (wild type for *pfdhfr*) and the W2 and V/1S strains (mutant type for *pfdhfr*) [16-18].

Genotyping for mutations of the *pfdhps *gene in codons 437, and 540 was performed by PCR of 35 cycles with 2 μl of DNA extract [15,19,14]. The PCR product obtained is 528 bp. The identification of mutations in these 2 codons was achieved by sequencing (ABI prism 310). The copy number for *pfmdr1 *gene was quantified by real-time PCR according to Sidhu *et al *and Price *et al *[20,21].

### Statistical analysis

Data analysis was performed using STATA^® ^9.0 (Stata Corporation, College Station, Texas, USA). Differences between proportions were compared using the Pearson chi-square or Fisher's exact test. Continuous variables were compared using the Wilcoxon test. The level of statistical significance retained was p < 0.05. Nine women were infected twice during pregnancy. Because of non-independent data, they were excluded from the analysis comparing the proportions of mutant parasites at different time-points during pregnancy.

## Results and discussion

**Figure 1:**
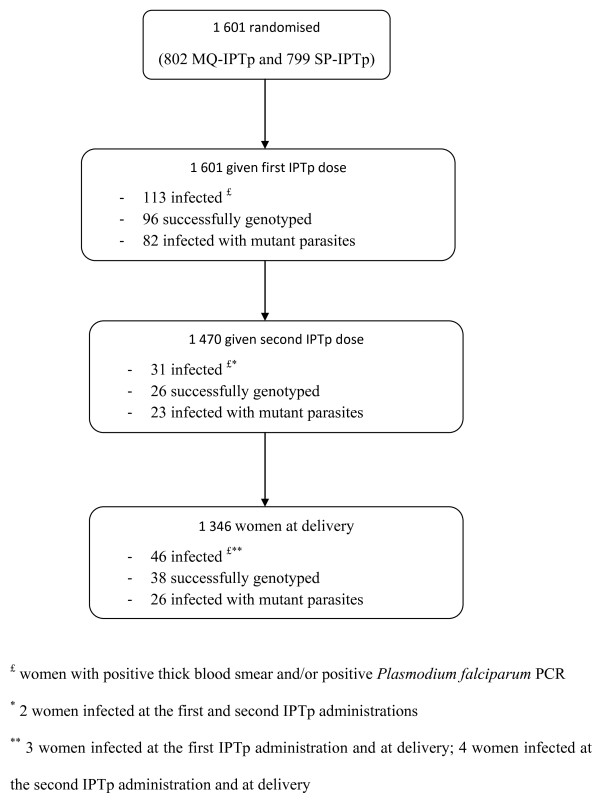
**Study profile**. IPTp, intermittent preventive treatment during pregnancy; SP, sulphadoxine-pyrimethamine; MQ, mefloquine.

A total of 181 women were infected during pregnancy, and among them nine were infected twice. They presented an average age of 23 years, and 41% were primigravidae. A total of 110 women had received SP, and 71 had received MQ. From the 190 samples collected and analysed, 160 genotyping were successful. A total of 113 samples collected at inclusion (before first IPTp dose), 31 at second IPTp administration (two months later in average), and 46 at delivery were investigated (Figure 1). A single copy of the *pfmdr1 *gene was found in parasites from all pregnant women at first and second IPTp administrations, as well as at delivery. This result indicates that the parasites present in these women were all of wild-type genotype for the *pfmdr1*gene.

At inclusion, the prevalence of triple *pfdhfr *mutant was 85% in parasites sampled from the 96 women, before any drug administration. Similar prevalence rates were observed in the MQ and SP groups (Table 1). Mutation at codons *pfdhfr *164 and *pfdhps *540 was not observed in any sample, thus no quintuple mutants were observed. The proportion of *pfdhfr *triple mutant parasites (51, 59, and 108) did not vary significantly at the 3 sampling times, neither in the SP group (80% vs. 92% vs. 68%; *P *= 0.33) nor in the MQ group (90% vs. 89% vs. 64%; *P *= 0.07) (Table 1). Similarly, the prevalence of quadruple mutations (at codons 51, 59, and 108 of the *pfdhfr *gene and codon 437 of the *pfdhps *gene) did not vary at the 3 sampling times (74% vs. 92% vs. 61%; *P *= 0.19 for the SP group, and 81% vs. 71% vs. 60%; *P *= 0.39 for the MQ group). Women having presented more than one infection during follow-up, and thus being included in more than one group, were excluded from those analyses to avoid data dependency.

**Table 1:** Prevalence rates of *pfdhfr*/*pfdhps *mutant parasites during the course of pregnancy, by treatment group.

Molecular marker	Before first IPTp administration	Before second IPTp administration	At delivery
		**SP**	

*pfdhfr *51	53/57	18/18	28/28
*pfdhfr *59	45/54	17/20	20/27
*pfdhfr *108	54/59	21/21	28/29
*pfdhfr *164	0/50	0/15	0/19
*pfdhfr *triple mutants*	43/53	16/18	19/27
*pfdhps *437	52/55	21/22	27/27
*pfdhps *540	0/62	0/22	0/28
*pfdhfr*/pfdhps quadruple mutants**	37/49	16/18	16/22

		**MQ**	

*pfdhfr *51	44/47	9/9	10/11
*pfdhfr *59	40/43	7/8	10/11
*pfdhfr *108	46/47	9/9	11/11
*pfdhfr *164	0/41	0/8	0/9
*pfdhfr *triple mutants*	39/43	7/8	8/11
*pfdhps *437	39/43	6/7	10/10
*pfdhps *540	0/47	0/8	0/9
*pfdhfr/pfdhps *quadruple mutants*	34/42	4/6	7/10

* *pfdhfr *triple mutants: (*pfdhfr *51, 59 and 108)

** *pfdhfr/pfdhps *quadruple mutants: *pfdhfr *51, 59 and 108 + *pfdhps *437

At delivery, the prevalence of triple or quadruple mutant parasites did not differ between the MQ and SP groups (64% vs 70%; *P *= 0.48; and 60% vs 65%; *P *= 0.54, respectively) (Table 1).

Such a high prevalence of mutations at inclusion, before any drug administration, is not surprising as prevalence rates above 50% of the triple *pfdhfr *mutant were also reported in Viet Nam [22], Malaysia [23], and Brazil [24]. In Africa, this rate ranges from 40% [25,26] to 75% [27,28]. These results in the context of IPTp are also similar to those published by Bouyou-Akotet *et al *[29], where the prevalence rates of triple and quadruple mutants were 80% and 53%, respectively. Such a high prevalence rate of mutations at inclusion heavily impairs any conclusion on the possible selection of parasite populations inducing an increase in resistance to SP. A very high number of infected pregnant women would be required for such an investigation. Even in areas of intense *P. falciparum *transmission, the expected proportion of women infected at enrolment may be relatively high (in the 15 to 25% range), but is drastically reduced (to a few percents) in women receiving IPTp. This study included 1601 pregnant women, a significant number of women. However, given the high prevalence of mutants at inclusion, the power to detect a limited increase of the prevalence of mutants during follow-up was too low. There was no difference in parasitaemia between women infected with mutant parasites compared to women with wild parasites neither at inclusion (median parasitaemia: 1408/mm3 vs. 1236/mm3, *P *= 0.89) nor at delivery in the SP group (median parasitaemia: 7714/mm3 vs. 15 743/mm3, *P *= 0.86). In addition, the genotyping results were not correlated with clinical efficacy of the drug, as assessed by placental infection or low birth weight (birth weight < 2,500 g) rates. The risk of placental infection did not differ in the absence or in the presence of triple and/or quadruple mutants during pregnancy in the SP group (0% (0/10) vs 2.4% (1/41); *P *= 0.81). Similarly, the risk of low birth weight baby did not differ in the absence or in the presence of triple or quadruple mutants during pregnancy. The mean weight of offspring of women infected during pregnancy (at inclusion or at second administration of IPTp) was 2724 g (2473-2975) if a triple or quadruple mutant was present, and 2808 g (2672-2944) if not (*P *= 0.29).

The women who were infected at delivery were often not the same as those previously infected by a triple or quadruple mutant parasite. In other words, women infected by a triple or quadruple mutant parasite during their pregnancy showed no increased risk for placental infection, as compared to women infected with wild type parasites. This suggests a lack of correlation between multiple *pfdhfr*/*pfdhps *mutations and SP failure in the context of IPTp, as assessed by the presence of a placental infection.

## Conclusion

The very high proportion of *pfdhfr*/*pfdhps *triple and quadruple mutations observed in this study (64 to 89% of infections for the three-time monitoring) did not allow to conclude statistically, although it is unlikely that infection with these genotypes actually correlates to the lack of efficacy of SP in the context of IPTp. Indeed, the current study shows that SP retained efficacy in preventing placental infection. Nevertheless, it is now obvious that SP will soon be compromised in whole Africa, and an urgent need exists to assess alternative drug regimens for IPTp, as well as to monitor SP efficacy for IPTp by other means than molecular markers [30]. Molecular markers have been recommended, but given the current prevalence of mutant parasites, their usefulness is questionable, and alternative strategies have to be developed.

## Competing interests

The authors declare that they have no competing interests.

## Authors' contributions

GB participated in the design of the study, supervised molecular studies and drafted the manuscript. VB provided the samples, performed the statistical analysis, and participated in writing the manuscript. DB carried out the molecular genetic studies. AC carried out the molecular genetic studies. AM participated in the design of the study. MC participated in the design of the study, and participated in writing the manuscript. PD participated in the study design and overall coordination, and finalized the manuscript. All authors read and approved the final manuscript.
